# Suppression of transforming growth factor-β effects in rabbit subconjunctival fibroblasts by activin receptor-like kinase 5 inhibitor

**Published:** 2010-09-16

**Authors:** Jennifer Sapitro, Jeffrey J. Dunmire, Sarah E. Scott, Vijay Sutariya, Werner J. Geldenhuys, Michael Hewit, Beatrice Y.J.T. Yue, Hiroshi Nakamura

**Affiliations:** 1 Department of Ophthalmology, Summa Health System, Akron, OH; 2 Department of Pharmaceutical Sciences, Northeastern Ohio Universities Colleges of Medicine and Pharmacy, Rootstown, OH; 3 Department of Behavioral and Community Health Sciences, Northeastern Ohio Universities Colleges of Medicine and Pharmacy, Rootstown, OH; 4 Department of Ophthalmology and Visual Sciences, University of Illinois at Chicago, College of Medicine, Chicago, IL

## Abstract

**Purpose:**

Transforming growth factor-β (TGF-β) activity has been implicated in subconjunctival scarring in eyes following glaucoma filtration surgery (GFS). The purpose of this study is to determine whether an inhibitor for activin receptor-like kinase (ALK) 5 (also known as TGF-β receptor type I) could suppress TGF-β activity and thereby promote filtering bleb survival after GFS in a rabbit model.

**Methods:**

An ALK-5 inhibitor, SB-505124, was used. A docking study was performed to investigate the interaction between the inhibitor and the receptor. Immunofluorescence for connective tissue growth factor (CTGF) and α-smooth muscle actin (α-SMA) was performed in cultured rabbit subconjunctival fibroblasts. Immunoblotting for phosphorylated Smad2 (pSmad2), CTGF, and α-SMA was also performed. In an in vivo rabbit GFS model, SB-505124 was delivered in a lactose tablet during surgery. Eyes were examined by slit-lamp and intraocular pressure (IOP) was measured until the time of bleb failure or up to 28 days after surgery. Tissue sections on day 5 after surgery were histologically evaluated after staining with hematoxylin and eosin. The sections were also immunostained for CTGF and α-SMA. In addition, cell outgrowth from dissected subconjunctival tissues placed in a cell culture flask with media was investigated.

**Results:**

The docking study indicated hydrogen bond interactions between SB-505124 and amino acids His-283 and Ser-280 of ALK-5. Suppression of pSmad2, CTGF, and α-SMA by SB-505124 was observed in cultured fibroblasts. Filtering blebs in the GFS with SB-505124 group were maintained for more than 10 days, and the period of bleb survival was significantly longer than that in controls. IOP levels after surgery seemed to be related to bleb survival. Histologically, subconjunctival cell infiltration and scarring at the surgical site in the GFS with SB-505124 and mitomycin C (MMC) groups were much subsided compared to controls. Suppression of CTGF and α-SMA by SB-505124 was also observed by immunofluorescence. Cell outgrowth from explants dissected from eyes to which SB-505124 was applied during GFS was robust while outgrowth was poor from those treated with MMC.

**Conclusions:**

The ALK-5 inhibitor SB-505124 was efficacious both in vitro and in vivo in suppressing the TGF-β action. The inhibitor may provide a novel therapy for preventing ocular inflammation and scarring.

## Introduction

Transforming growth factor-β (TGF-β), a family of structurally related multifunctional cytokines, has a wide range of biologic functions including cell growth, differentiation, apoptosis, and fibrogenesis [1-3]. TGF-β typically is secreted in a latent form and is activated through a complex process of proteolytic activation and dissociation of latency protein subunits [4,5]. TGF-β has emerged as a key mediator of the fibrotic response to wounding. It is upregulated during different types of wound healing in tissues including the eye, liver and skin [3,6-8]. In the eye, TGF-β has been shown to be important in scarring in conditions such as proliferative vitreoretinopathy [9], cataract formation [10], corneal opacities [11], and choroidal neovasculaization [12,13] as well as in subconjunctival scarring, a complication of filtration surgery in glaucoma [14,15]. TGF-β1 and TGF-β2 are expressed in the filtering bleb after glaucoma filtration surgery (GFS) while TGF- β2 is the predominant form in the aqueous humor [16,17].

In GFS, postoperative scarring at the wound site is a critical determinant of the surgical outcome [18,19]. Although anti-scarring agents such as mitomycin C (MMC) and 5-fluorouracil can prevent post-operative scarring and improve surgical outcome [20,21], they cause widespread fibroblast cell death and are associated with severe and potentially blinding complications [22,23]. The central role of TGF-β in wound repair has led to other strategies [13] such as the use of anti-TGF-β antibody [24,25] and antisense oligonucleotides/siRNA [26,27] to block the TGF-β action. A monoclonal antibody for TGF-β2, called CAT-152, which neutralizes TGF-β function, was investigated as an adjunct in preventing scar formation following GFS. However, in a phase III clinical trial, there was no difference between CAT-152 and a placebo in preventing the failure of primary trabeculectomy in human glaucoma eyes [28]. Antisense oligonucleotides and siRNA for TGF-β or TGF-β receptors type II have also been investigated for silencing of the gene expression [26,27]. Although their ability to suppress scarring was reported in vitro and in vivo, their working period and stability may not be suitable for clinical applications. Moreover, there may also be complications from a general knockdown of gene expression.

Receptors and kinases are generally believed to be effective targets for selectively blocking signaling pathways in a variety of biologic systems [29,30]. Members of the TGF-β superfamily, i.e., TGF-βs, activin, myostatin, and bone morphogenetic proteins, bind to type I and type II serine/threonine kinase receptors and transduce intracellular signaling through Smad proteins. There are seven known mammalian type I receptors, activin receptor-like kinase (ALK) 1–7, and five type II receptors [31,32]. Unique combinations of the type I and type II receptors confer specificity of ligand signaling. TGF-βs display a high affinity for the type II receptors and do not interact with the isolated type I receptors [4,33]. Signal transduction of TGF-βs is initiated by binding to type II receptor, followed by its association with ALK-5 (also called TGF-β receptor type I). The activated ALK-5 in turn phosphorylates and activates transcription factors Smad2/3 [4,5]. TGF-β type II receptor and ALK-5 should therefore be reliable targets to block the TGF-β signaling pathway.

Recently, ALK-5 inhibitors, that selectively block ALK-5 as well as ALK-4/7, which share similarity with ALK-5 in their kinase domains [32], have been described in several publications to be effective suppressors of TGF-β activity [34-41]. In experiments described herein, the ALK-5 inhibitor SB-505124 induced a marked reduction in levels of phosphorylated Smad2 (pSmad2) and downstream proteins of TGF-β, connective tissue growth factor (CTGF) and α-smooth muscle actin (α-SMA), in cultured rabbit subconjunctival fibroblasts. In addition, in an in vivo rabbit GFS model, it was shown that SB-505124 extended the period of filtering bleb survival and its toxicity was substantially lower than that of MMC.

## Methods

### Docking study

The interaction between SB-505124 and the kinase ALK-5 was investigated using computational docking software. The starting point for this study was to use the well known GOLD docking software (version 4.1; Cambridge Crystallographic Center, Cambridge, UK), which uses a genetic algorithm for finding a docking pose, i.e., mode of binding of compound to the protein. The protein X-ray crystal structure of ALK-5, 1VJY.pdb, was downloaded from the Protein Data Bank and prepared for the docking study, using the standard protocols which are set-up in GOLD. These include separation of the co-crystallized ligand and protein, as well as addition of the hydrogens to the protein, which are omitted in the protein databank files. SB-505124 was computationally drawn using MarvinSketch (version 3.5.4). Before this compound could be docked into ALK-5 using GOLD, it had to be rearranged in 3-D to find a biologically suitable starting point, which is that of an energy minimized form of the compound. To do this, MOE (Chemical Computing Croup, Montreal, Canada) was used, with the following set-up: force-field was MMFF94, termination was 0.001 kcal/mol. Using the kinase scoring function in GOLD, a suitable docking pose was identified for SB-505124 with ALK5.

### Cell culture

Fresh eyes, including intact conjunctiva with palpebral margins, enucleated from New Zealand White (NZW) rabbits were obtained from Pel*-*Freez Biologicals (Rogers, AR). Subconjunctival fibroblasts were cultured from subconjunctival tissues, called tenon’s membrane, dissected from rabbit eyes. The cells were maintained in serum-containing media and were passaged as previously described [42]. Third to fifth passages of cells were used for experiments.

### Treatment with TGF-β2, inhibitor and MMC

Fibroblasts were pre-treated with various concentrations of ALK inhibitor SB-505124 for 1 h and were then incubated for 48 h after 2 ng/ml of TGF-β2 (R&D Systems, Minneapolis, MN) was added. Controls that were not treated with the inhibitor but were treated with or without TGF-β2 were also prepared. To examine whether SB-505124 causes cell death, cells (100% confluent) in 12-well plates were pre-treated with SB-505124 (10 µM) for 1 h and were then incubated for 48 h after 2 ng/ml of TGF-β2 was added. Controls that were treated with 0.04% MMC (Kyowa Hakko Kirin, Tokyo, Japan) were also examined. Cells were treated in 0.04% MMC for 5 min and were then incubated with 2 ng/ml of TGF-β2 for 48 h after MMC removal. The number of cells was counted using a hemocytometer (Hausser Scientific, Horsham, PA) after trypsinization.

### Western blotting

After treatment, fibroblasts were lysed in a RIPA lysis buffer (20 mM Tris, 150 mM NaCl, 1 mM EDTA, 1% Triton X-100) for blotting of pSmad2 or in a lysis buffer (CelLytic M Cell Lysis Reagent; Sigma, St. Louis, MO) for blotting of CTGF and α-SMA. Equal amounts of protein (20 μg/lane) were resolved on 10% sodium dodecyl sulfate (SDS)-polyacrylamide gels. The proteins were then transferred to PVDF membranes (Bio-Rad Laboratories, Hercules, CA) for probing with monoclonal rabbit anti-pSmad2 (1:500; Millipore, Billerica, MA) followed by HRP-conjugated goat anti-rabbit IgG (1:20,000; Santa Cruz Biotechnology, Santa Cruz, CA), monoclonal rabbit anti-Smad2 (1:500; Invitrogen, Carlsbad, CA) followed by HRP-conjugated goat anti-rabbit IgG (1:25,000; Santa Cruz Biotechnology), polyclonal goat anti-CTGF (1:1000; Santa Cruz Biotechnology) followed by HRP-conjugated donkey anti-goat IgG (1:10,000; Jackson ImmunoResearch, West Grove, PA) or monoclonal mouse anti-α-SMA (1:4,500; Sigma) followed by HRP-conjugated goat anti-mouse IgG (1:100,000; Jackson ImmunoResearch). The signal was detected by enhanced chemiluminescence. In CTGF and α-SMA blotting, the blots were also probed with HRP-conjugated monoclonal mouse anti-β-Actin (1:50,000; Sigma) or mouse anti-GAPDH (1:2,500; Novus Biologicals, Littleton, CO) followed by HRP-conjugated goat anti-mouse IgG (1:75,000) to control for equal protein loading. Densitometry was performed using Kodak Molecular Imaging Software version 4.5 (Carestream Health, Rochester, NY). The band intensity of pSmad2, CTGF or α-SMA was normalized against that of Smad2, β-actin, or glyceraldehyde 3-phosphate dehydrogenase (GAPDH).

### Immunocytochemistry

Subconjunctival fibroblasts were cultured on 8-well chamber slides. After inhibitor and TGF-β2 treatment, fibroblasts were fixed with 4% paraformaldehyde and permeabilized. Cells were then incubated with polyclonal goat anti-CTGF (1:100) followed by Alexa Fluor donkey anti-goat IgG (10 µg/ml; Invitrogen) or monoclonal mouse anti-α-SMA (1:400) followed by Alexa Fluor goat anti-mouse IgG (10 µg/ml). Slides were mounted with aqueous mounting medium with 4’,6-diamidino-2-phenylindole (DAPI) and viewed by fluorescence microscopy.

### Rabbit GFS model

The experimental protocol was approved by the Institutional Animal Care and Use Committee at Northeastern Ohio Universities Colleges of Medicine and Pharmacy. Animal care guidelines comparable to those published by the US Public Health Service were followed. NZW rabbits were anesthetized by subcutaneous injection of a combination of medetomidine (approximately 0.25–0.5 mg/kg; Pfizer Animal Helath, New York, NY) and ketamine (15 −20 mg/kg; Fort Dodge Animal Health, Fort Dodge, IA). Additional injections (one fourth to one half of the original dose) were also given every 30 to 45 min for maintenance. Local anesthesia was provided with proparacaine HCl (0.5% eye drops; Alcon, Fort Worth, TX). After washing the eye for surgery with providone-iodine topical antiseptics (1:16 dilution with sterile water; Aplicare, Meriden, CT), all procedures were performed under sterile conditions. For the surgery, the eyelids were retracted using a speculum. A partial-thickness corneal traction suture (8–0 silk; Alcon) was placed in the superior cornea to rotate the eye inferiorly. A clear corneal paracentesis tract was made between the 12 and 2 o’clock positions, and Viscoelastic material (0.1–0.2 ml, Discovisc^®^; Alcon) was injected into the anterior chamber to maintain chamber form. Surgery was performed at the anterior, temporal and superior sites of eyes. A fornix-based conjunctival flap was raised behind the limbus. A scleral tunnel to the corneal stroma was then fashioned. A 22-G/25-mm venflon 2 cannula (Becton Dickinson, Franklin Lakes, NJ) was passed through the sclera until it was visible in the cornea. After entry of the cannula into the anterior chamber, the cannula was fixed to the scleral surface with a 10–0 nylon suture (Alcon). The conjunctival incision was closed by interrupted and mattress sutures using 9–0 nylon with taper cut needle (Ethicon, Somerville, NJ). One drop of 1% atropine and a single application of combined neomycin and dexamethasone ointment were applied to the ocular surface at the end of surgery.

GFS with MMC treatment was performed as a control (MMC control). Surgical sponges immersed with 0.04% MMC (Bedford Laboratories, Bedford, OH) were applied in the subconjunctival space at the surgical site for 5 min right after a fornix-based conjunctival flap was raised. The site was then washed with 500 ml of balanced salt solution.

### Delivery of SB-505124

Tablets containing 5 mg of SB-505124 and 65 mg lactose (6 mm in diameter, 1.0 mm in thickness) were prepared using a compression technique [43]. Prior to sutures of the conjunctival incision in GFS, the lactose tablet was placed on the sclera at the surgical site after being broken into several pieces. GFS without any adjuncts (no adjunct control) and GFS with lactose tablets devoid of inhibitors (tablet control) were used as controls.

### Eye examination after GFS

All animals were examined by slit-lamp daily during the first week after GFS, and at least twice a week thereafter until the time of an occurrence of bleb failure or post surgical infection, or up to 28 days post surgery. Filtering bleb, anterior chamber activity and depth, conjunctival hyperemia, and aqueous humor leakage were examined. Bleb failure was defined as the appearance of a flat, vascularized, scarred bleb in association with a deep anterior chamber. The lack of aqueous humor drainage into the bleb promoted by eye massage assisted in the judgment of bleb failure. Filtering blebs were also photographed with a digital camera (FinePix F40fd; Fujifilm, Tokyo, Japan).

IOP in both treated and non-surgically-treated eyes was measured with a TONO-PEN AVIA^®^ (Reichert Ophthalmic Instruments, Depew, NY) by gently touching the cornea after topical anesthesia with 0.5% proparacaine at least twice a week until the time of an occurrence of bleb failure or post surgical infection, or up to 28 days post surgery. The IOP reading was omitted if its confidence interval was less than 95%. An average of three measurements was taken to deduce IOP.

### Histological examination and immunofluorescence

Five days after surgery, rabbits were euthanized with Fatal-plus™ (Vortech Pharmaceutical, Dearborn, MI) following manufacturer’s instructions. Rabbit eyes were enucleated with palpebral margins to keep conjunctiva epithelium and subconjunctival space intact. Enucleated eyes were fixed with 10% buffered formalin, and 5 μm thick paraffin sections were prepared. The sections were stained with hematoxylin and eosin (H&E) for histological examination. For fluorescence staining, the sections were incubated at room temperature with goat anti-CTGF (1:50) followed by Alexa Fluor 594 rabbit anti-goat IgG (1:100; Invitrogen) and with mouse anti-α-SMA (1:100) followed by DyLight 488 goat anti-goat IgG (1:100; Jackson ImmunoResearch). Images were captured using an Olympus DX51 microscope and DP controller (Olympus, Tokyo, Japan).

### Subconjunctival tissue fibroblast outgrowth assay

To investigate if the efficacy of ALK-5 inhibitor in suppression of ocular scarring is related to its toxicity like MMC, a subconjunctival tissue fibroblast outgrowth assay was performed [44]. Five days after GFS with inhibitor or MMC, rabbits were euthanized as described above. Under an ophthalmic surgical microscope, subconjunctival tissues were dissected from the surgical site and 180° (6-o’clock position) from the surgical site (180° control). Each biopsy specimen was placed in a 25 cm^2^ cell culture flask with complete media. Care was taken in handling the biopsy specimens, in particular to ensure that the samples did not dry out and affect the cellular viability. The cell outgrowth from each explant was observed, and the length of the outgrowth in 4 quadrants was measured.

### Statistical analysis

Bonferroni multiple comparison was used to analyze cell numbers treated with SB-505124 and MMC treatments in vitro. Two-way ANOVA was performed to evaluate the fibroblast outgrowth from subconjunctival tissues. The Kaplan–Meier method was used to analyze bleb survival, and the samples that had post surgical infection were treated as censoring in the method. All statistical analyses were conducted by SPSS (ver.16; Chicago, IL).

## Results

### Interaction between SB-505124 and ALK-5

**Figure 1:**
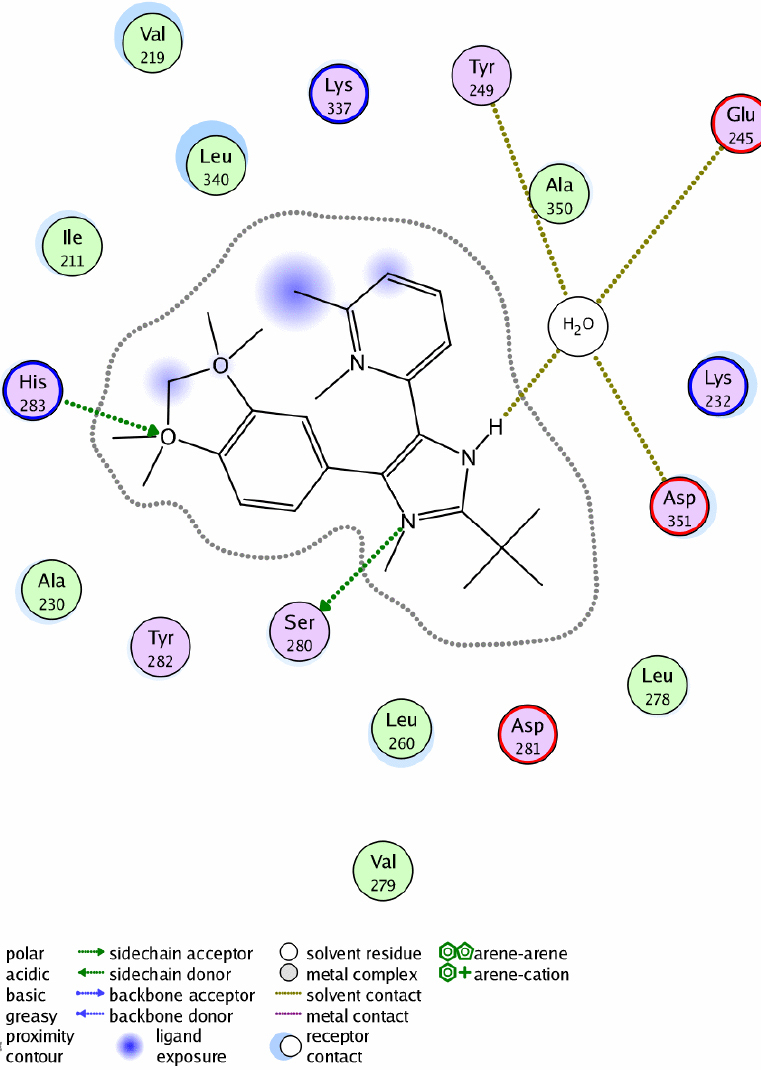
Docking results of SB-505124 (atom colors) in the ALK-5 ATP-binding pocket. The main interactions between SB-505124 and ALK-5 were seen to be between His-283 and Ser-280, which is located at the ATP binding site in the intracellular ALK-5 kinase domain. Additionally, a water molecule seems to form a hydrogen-bonding bridge between SB-505124 and amino acids Asp-351, Glu-245 and Tyr-249. Also shown is the overlay of a 1,5-naphthyridine compound which is (in green) co-crystallized with ALK-5.

To explore the interaction or binding of SB-505124 with ALK-5, we docked SB-505124 into the ATP/ligand binding pocket of ALK-5 (Protein data bank access code 1VJY) [45]. As can be seen from Figure 1, hydrogen bond interaction appears to be between SB-505124 and amino acid residues His-283 and Ser-280 of ALK-5 (Figure. 1), which is located at the ATP binding site in the intracellular ALK-5 kinase domain [46]. This binding mode of SB-505124 with ALK-5 appears to be similar to that of the co-crystallized ligand of 1VJY [45], a human expressed ALK-5 protein crystal. A water molecule is seen forming a hydrogen-bonding bridge between SB-505124 and amino acid residues Asp-351, Glu-245 and Tyr-249.

### SB-505124 impairs Smad2 phosphorylation and CTGF and α-SMA expression in vitro

**Figure 2:**
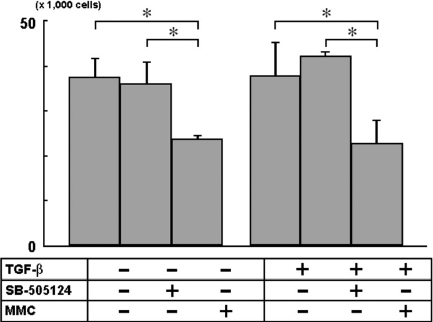
Effects of SB-505124 on cell density. Rabbit subconjunctival fibroblasts were incubated with 10 µM of SB-505124 or 0.04% MMC and then with or without TGF-β2 (2 ng/ml) in 12-well plates for 48 h (n=3).The number of cells was counted using a hemocytometer after trypsinization. A reduction in cell number or cell loss was not observed with or without TGF-β2 in the SB-505124, but was seen in the MMC treatment group . Asterisks indicate significant difference (p<0.05, Bonferroni multiple comparison).

**Figure 3:**
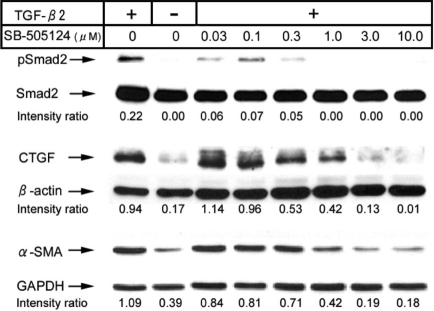
Western blotting for phosphorylated Smad2 (pSmad2), CTGF, and α-SMA. Rabbit subconjunctival fibroblasts were incubated with TGF-β2 and various concentrations of SB-505124. SB-505124 effectively reduced the pSmad2 level and the expression of CTGF and α-SMA induced by TGF-β2 in a concentration-dependent fashion. SB-505124 was effective when its concentration reached 1 µM, and the expression of CTGF and α-SMA at 3 and 10 μM of concentrations is below that in cells without the TGF-β2 stimulation. The blots were also probed for Smad2, β-actin, or GAPDH. The band intensity of pSmad2, CTGF and α-SMA was normalized against that of Smad2, β-actin, and GAPDH, respectively, and the ratios are shown.

**Figure 4:**
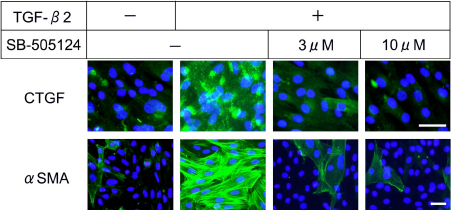
Immunofluorescence staining for CTGF and α-SMA in subconjunctival fibroblasts. Immunoreactive products were visualized with Alexa Fluor (green) labeling. Nuclei were stained with DAPI (blue). Rabbit subconjunctival fibroblasts were treated with or without TGF-β2 and/or SB-505124. A dramatic increase in staining for TGF- β2-induced proteins CTGF and α-SMA was observed following TGF-β2 incubation. The staining intensity of these proteins was markedly reduced when cells were treated concomitantly with SB-505124. The cell density was not affected by SB-505124 treatment. Bar, 50 µm.

With or without TGF-β2, SB-505124 treatment for 48 h did not result in cell loss while MMC treatment caused a significant (p<0.05) reduction in cell number (Figure 2). Western blotting for pSmad2, CTGF, and α-SMA was performed to examine the effect of ALK-5 inhibitor SB-505124 on downstream effects induced by TGF-β2. The levels of pSmad2, CTGF, and α-SMA were found to be reduced in a concentration-dependent fashion (Figure 3). The band intensity for each protein in samples treated with inhibitor was lower than that without inhibitor treatment under TGF-β2 stimulation. The reduction in protein levels was observed when the inhibitor concentration reached 1 μM, and the reduction of CTGF and α-SMA levels was found to be more pronounced with higher concentrations (3 and10 μM). By immunocytofluorescence, a dramatic increase in staining for CTGF and α-SMA was observed following TGF-β2 incubation (Figure 4). The staining intensity of TGF-β2-induced proteins was greatly reduced when cells were treated concomitantly with SB-505124.

### Bleb survival and IOP reduction after GFS in rabbit model

**Figure 5:**
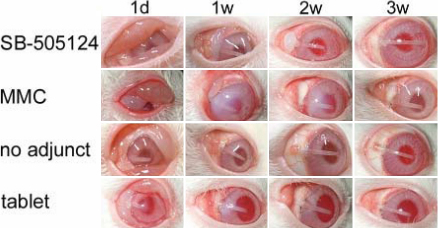
Appearance of filtering blebs after glaucoma filtration surgery (GFS). The first row; GFS with SB-505124, the second row; MMC control, the third row; no adjunct control and the bottom row; tablet control. In rabbits that received GFS with SB-505124 or MMC treatment, filtering blebs were maintained for 1 week after GFS and beyond whereas blebs in the no adjunct and tablet controls failed within 1 week. The blebs treated with SB-505124 failed by 3 weeks after surgery while the blebs in the MMC control were maintained.

**Figure 6:**
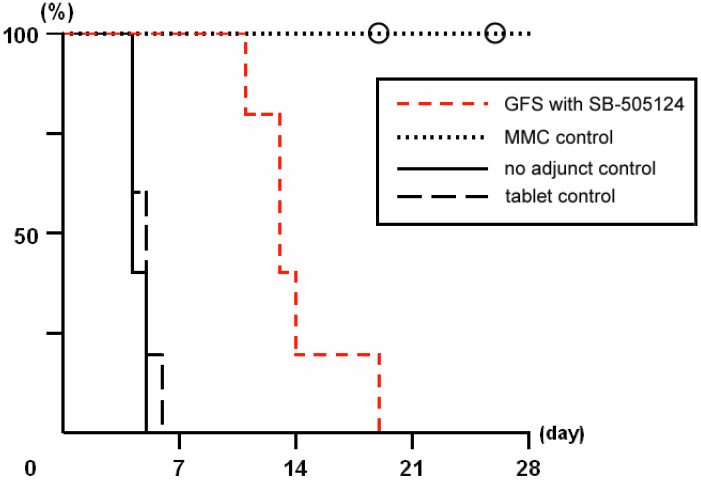
Survival curves of filtering blebs after glaucoma filtration surgery (GFS). Filtering blebs in the no adjunct (n=5) and the tablet (n=5) controls failed within 1 week. Treatment with SB-505124 (n=5) markedly increased the bleb survival period. In the MMC control (n=5), three blebs survived during the observation period, but post surgical infection occurred in filtering blebs of the remaining two on days 19 and 26, which are indicated by circles. A Kaplan–Meier analysis showed a significant difference in the survival distributions among the four comparison groups (log rank=25.688, p<0.01).

Bleb appearance after GFS and survival curves of blebs are shown in Figure 5 and Figure 6, respectively. On post surgery day 1, large filtering blebs were observed in all treated eyes. A flat or shallow anterior chamber was seen in four of five eyes in SB-505124 and MMC groups and one of five eyes in no adjunct control group, but aqueous humor leakage was not detected in any cases by the Seidel test. The filtering blebs in the GFS with SB-505124 group were maintained for more than 10 days without any severe postoperative complications, and the blebs were clearly observed. In the MMC control group, three of five blebs survived during the observation period, while post surgical infection occurred in filtering blebs of the remaining two on days 19 and 26. Blebs in the no adjunct and tablet controls failed within 1 week. A Kaplan–Meier analysis showed a significant difference in the survival distributions among the four comparison groups (log rank=25.688, p<0.01).

**Figure 7:**
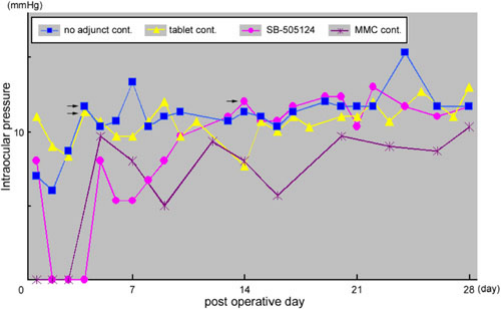
Intraocular pressure (IOP) after glaucoma filtration surgery (GFS). IOP measurements after GFS in a typical case of each treatment group, i.e., no adjunct control, tablet control, GFS with SB-505124 and MMC control groups, are shown. The lowest IOP was noticed on post-GFS day 2 or 3 in each case. IOP in the no adjunct control and tablet control groups exceeded 10 mmHg before week 1 and subsequently plateaued. IOP in the GFS with SB-505124 groups exceeded 10 mmHg before week 2. IOP in the MMC control is relatively low throughout the observation period. The IOP readings seem to be related to bleb survival in each case. Arrows indicate the occurrence of bleb failure.

IOP measurements from a typical case in each rabbit group and the average IOP after GFS are shown in Figure 7 and Table 1, respectively. In all treatment or control groups, the average IOP on day 1 after GFS was lower than 10 mmHg. The IOP was lower than 10 mmHg at week 1 in SB-505124 and MMC groups whereas it exceeded 10 mmHg at week 1 and thereafter in no adjunct and tablet controls. The IOP in SB-505124 group exceeded 10 mmHg at week 2. IOP in the MMC control group was relatively low throughout the entire observation period. The IOP readings after GFS seemed to reflect bleb survival in each group.

**Table 1:** Average of IOP after GFS in each group.

**GFS group**	**Day 1**	**Week 1**	**Week 2**	**Week 3**	**Week 4**
No adjunct control	8.2±1.0 (4)	12.1±1.2 (5)	10.3±1.6 (4)	11.3±0.6 (3)	12.0±0.3 (3)
Tablet control	9.7±1.3 (5)	10.1±0.4 (5)	10.2±2.0 (4)	11.5±0.7 (2)	13.0±0.0 (2)
SB-505124	5.0±4.6 (5)	4.5±4.6 (5)	13.2±3.3 (4)	12.8±3.5 (2)	13.0±1.9 (2)
MMC control	4.5±4.4 (5)	8.0±1.5 (5)	9.4±2.0 (5)	10.6±0.9 (4)	9.6±1.1 (4)

Average±standard deviation of IOP (mmHg) was shown at each time point. Sample numbers were shown in parentheses.

### Histological examination and immunofluorescence

**Figure 8:**
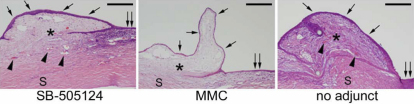
Hematoxylin and eosin staining in tissue sections from eyes five days after surgery. Infiltration of only a few inflammatory cells and mild scarring were observed in the subconjunctival space of eyes in the GFS with SB-505124 (left panel) or the MMC control (center panel) group, whereas numerous inflammatory cells and massive scarring were seen in the no adjunct controls (right panel). At the corneal limbus, infiltration of cells was observed in all 3 groups. Thinner conjunctival epithelium was seen in the MMC control compared to that in the GFS with SB-505124 and no adjunct control groups. Subconjunctival blood vessels were noted in GFS with SB-505124 and no adjunct control groups in general, but seldom in the MMC control group. Arrows indicate the conjunctival epithelium; double arrow, the limbus; arrowhead, the subconjunctival blood vessel; asterisk, the subconjunctival space; and S, the sclera. Bar, 200 μm.

**Figure 9:**
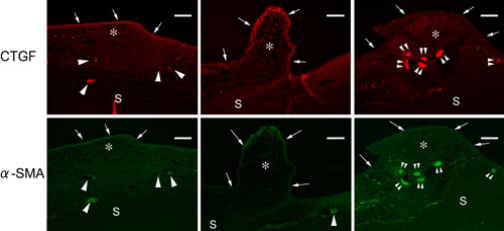
Immunofluorescence for CTGF and α-SMA in tissue sections from eyes five days after glaucoma filtration surgery (GFS). The top row; immunofluorescence staining for CTGF (red), the bottom row; immunofluorescence for α-SMA (green). Diffuse subconjunctival cell staining for both CTGF and α*-*SMA was observed in the no adjunct control while weaker and more scarce staining was demonstrated in the SB-505124 and MMC groups. The weaker and less staining seems due to suppression of CTGF/α*-*SMA expression in the SB-505124 group. In the MMC group, it is based on a lower cell number. Arrows indicate the conjunctival epithelium; asterisk, the subconjunctival space; S, the sclera; arrowhead, blood vessel with red blood cells; and double arrowhead, non specific staining. Bar, 100 μm.

As shown with H&E staining (Figure 8), infiltration of only a few inflammatory cells and slight scarring were observed in the subconjunctival space of eyes in the GFS with SB-505124 and the MMC control groups. By contrast, numerous inflammatory cells and massive scarring were seen in the no adjunct control. At the limbus, infiltration of cells was observed in all types of GFS. The conjunctival epithelium appeared to be thinner in the MMC control group compared to that in the GFS with SB-505124 and the no adjunct control groups. Subconjunctival blood vessels were noted in the GFS with SB-505124 and the no adjunct control groups, but seldom in the MMC controls. Diffuse immunofluorescence staining for both CTGF and α*-*SMA was detected in cells at subconjunctival area in the no adjunct controls. The staining was weaker and scarce in the SB-505124 and MMC groups (Figure 9).

### Subconjunctival fibroblast outgrowth assay

**Figure 10:**
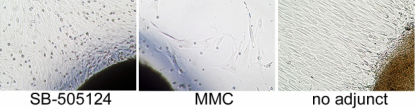
Fibroblast outgrowth from subconjunctival tissues. Five days after GFS, subconjunctival tissues were dissected, and each biopsy specimen was placed in a 25 cm^2^ cell culture flask with complete media. Image of fibroblast outgrowth from subconjunctival tissues at day 10 in culture is shown. Cell outgrowth from explants of the GFS with SB-505124 (left panel) and the 180° control (right panel) groups is robust whereas outgrowth is poor from that of the MMC control group (center panel). A few small, round specks that appeared to be dead cells were found on top of the outgrowth from tissue treated with inhibitor and MMC, but seldom in that from the 180° control tissues. This suggests that, while its toxicity is vastly reduced compared to MMC, 5 mg of SB-505124 delivered within a lactose tablet is not entirely nontoxic.

**Figure 11:**
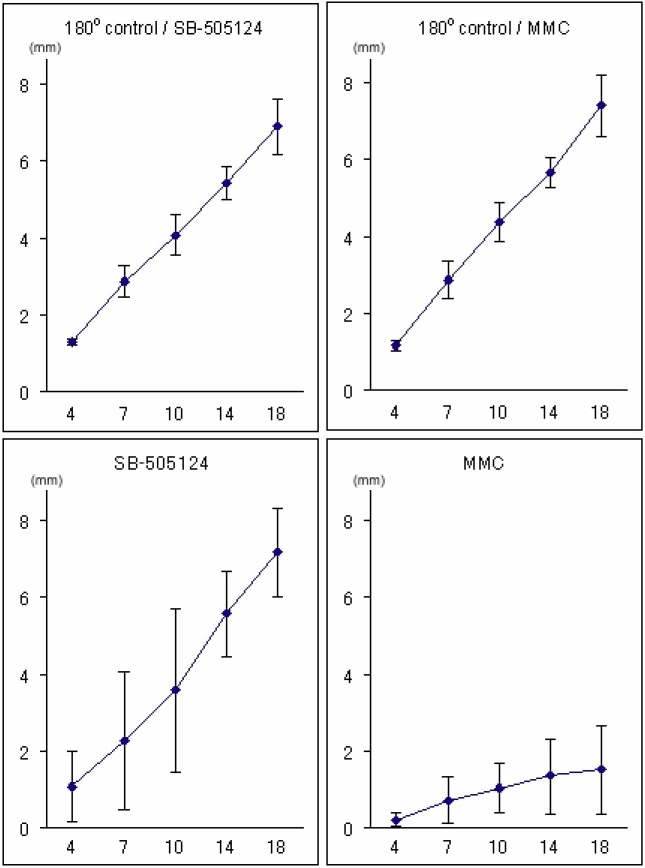
Line graph for cell outgrowth. For each of the GFS with SB-505124 or MMC groups, subconjunctival tissues were dissected from eyes of two rabbits that underwent GFS five days after surgery. Three pieces of subconjunctival tissue were dissected from the surgical site and 180° (6-o’clock position) from the surgical site (180° control). The cell outgrowth from each explant was observed, and the length of the outgrowth from the explant in four quadrants was measured. While a significant difference was observed in cell growth between the surgical site specimen and the 180° control (p<0.01, 2-way ANOVA) in GFS with MMC, there was no significant difference in that with SB-505124. Bars indicate standard deviation.

Cell outgrowth from the subconjunctival tissue from GFS eyes treated with SB-505124 was robust while it was poor from those treated with MMC (Figure 10). There was no significant difference in cell outgrowth between subconjunctival tissues treated with SB-505124 and the 180° controls whereas a significant difference was noted between MMC and controls (p<0.01, 2-way ANOVA, Figure 11). A few small, round specks that appeared to be dead cells were noted on top of the outgrowth from tissues treated with inhibitor and MMC, but seldom in that from the 180° control tissues.

## Discussion

The present study demonstrates the efficacy of an ALK-5 inhibitor, SB-505124, in the possible treatment of filtration surgery in glaucoma. SB-505124 is shown to block TGF-β-mediated induction of pSmad2, CTGF, and α-SMA in cultured rabbit subconjunctival fibroblasts in a dose-dependent fashion by both western blotting and immunofluorescence staining inhibition. The inhibition effect is observed when the inhibitor concentration reaches 1 μM. The expression of downstream proteins in cells treated with 3 and 10 μM of the inhibitor is below that in cells without the TGF-β2 stimulation, suggesting that 3 μM of SB-505124 is sufficient to block the TGF-β2 function. Our docking studies indicate that the affinity of SB-505124 with ALK-5 is likely due to hydrogen bonding between the two molecules, similar to other ALK-5 kinase inhibitors [47].

In a previous paper, SB-505124 was reported to be a potent inhibitor of the in vitro kinase activity of ALK-5 for its substrate Smad3 with an IC_50_ of 47±5 nM [37], which is much lower than the effective concentration in our study. The disparity may be related to differences in cell types, species, experimental methods and parameters studied.

The TGF-β pathway implicated in scarring has been investigated as an approach to reduce severe post-surgical complication caused by GFS with MMC. While promising preliminary results had been obtained, a monoclonal antibody for TGF-β2 (CAT-152) failed to achieve significant improvement in preventing the failure of primary trabeculectomy in human glaucoma eyes [28]. It is speculated that CAT-152 might have been underdosed [28]. Recently, another ALK-5 inhibitor, SB-431542, was reported to be able to abrogate TGF-β induced upregulation of α-SMA, CTGF and collagen type I at concentrations of 1–20 μM in vitro [41]. However, subconjunctival injection of 0.1 ml of 0.5 or 2 mM SB-431542 immediately after the surgery and on days 1, 2, 3, and 7 after surgery did not result in longer survival of filtering blebs although the conjunctiva was more translucent compared to controls.

During the initial phase of our study, we examined several ALK-5 inhibitors, including 616451 [36], 616452 [38] (Calbiochem, San Diego, CA), A-83–01 [40], SB-431542 [34], SB-505124 [37], and SB-525334 (Sigma) [48] in rabbit GFS. These inhibitors were delivered by subconjunctival injection of 0.1 ml solution with up to 300 μM concentration (during surgery and twice a day up to 5 day after surgery) and by tablet placement containing 1 mg of a given inhibitor. Only a slight improvement was observed with the use of tablets containing SB-505124 (data not shown). In subsequent experiments, 5 mg of SB-505124 was tested, and successfully improved bleb survival. The dosage of the inhibitor used thus seems to be of crucial importance.

By H&E staining, the conjunctival epithelium and subconjunctival blood vessels in GFS eyes treated with SB-505124 appear undamaged, similar to that in the no adjunctive controls. Infiltration of a few inflammatory cells and mild scarring were observed. In the MMC control group, infiltration and scarring were also at a low level, but the conjunctival epithelium became thinner and few blood vessels were seen. Additionally, the subconjunctival fibroblast outgrowth assay revealed that the cell outgrowth from subconjunctival tissues treated with SB-505124 was much more robust compared to that from tissues treated with MMC. Meanwhile, suppression of CTGF and α-SMA by SB-505124 after GFS was observed by immunofluorescence. Overall, our results indicate that SB-505124 is able to suppress subconjunctival scarring, via blocking of the TGF-β activity, in an in vivo rabbit model without much tissue damage and with vastly reduced toxicity compared to MMC.

In some cases, especially in the no adjunct control and tablet control groups, IOP reduction in operated eyes was limited, and IOP in the early post surgery period was higher than that in other reports [41]. In our GFS, viscoelastic material was injected into the anterior chamber to maintain chamber form during surgery. Therefore, even in the early post-surgical period, for example day 1 after GFS, dramatic IOP reduction was not seen in all cases (Figure 9) presumably because of the viscoelastic material that remained in the anterior chamber.

Our data also showed that while bleb survival was improved by SB-505124, the survival period was shorter that that produced by MMC. It is speculated that maintaining an appropriate concentration of SB-505124 in the filtering bleb may be the key to extend the period of bleb survival. Further investigation employing a controlled drug delivery system needs to be conducted.

In summary, the current study demonstrates that blocking the TGF-β signaling pathway by local application of SB-505124 prevents ocular scarring after GFS. Our results underscore the potential of a novel therapy for GFS and for preventing the inflammatory response and scarring in ocular diseases. This approach may also have applications for other surface tissues, including the skin.
